# Chronic Illnesses: Varied Health Patterns and Mental Health Challenges

**DOI:** 10.3390/healthcare13121396

**Published:** 2025-06-11

**Authors:** Ângela Leite

**Affiliations:** Centro de Estudos Filosóficos e Humanísticos, Universidade Católica Portuguesa, 4710-362 Braga, Portugal; aleite@ucp.pt

**Keywords:** chronic illnesses, hypertension, diabetes, cancer, health behaviors, psychological well-being

## Abstract

**Background/Objectives:** Hypertension, diabetes, and cancer are three prevalent chronic conditions with distinct etiologies and significant global health impacts. This study aimed to explore the diverse impacts of different chronic illnesses on health behaviors and psychological well-being, with a focus on identifying and addressing the unique challenges faced by individuals with hypertension, diabetes, and cancer. It was hypothesized that health behaviors and psychological well-being would differ significantly among individuals with hypertension, diabetes, and cancer, reflecting the distinct demands and psychosocial impacts of each condition. **Methods**: The database of Americans’ Changing Lives, Wave 6, including 767 participants, was used (56.1% hypertension, 20.8% diabetes, and 19.9% cancer cases). Variables concerning physical and mental health issues were chosen. Descriptive statistics summarized the data. Chi-squared and t-tests assessed associations and group differences, with effect sizes reported. Logistic regression examined predictors of hypertension, diabetes, and cancer. Sensitivity analyses excluded outliers. **Results**: Hypertensive individuals are more likely to show cognitive impairment and unhealthy behaviors, including poor self-rated health, higher BMI, lower physical activity, and altered alcohol use. Risk increases with age, widowhood, retirement, hospital admissions, and poor mental health, while more emergency room or doctor visits slightly reduce it. People with diabetes experience greater depressive symptoms, hopelessness, and financial stress. They also tend to have poorer self-rated health, higher BMI, and less physical activity. Risk is higher for separated individuals and lower for females. Psychological distress is a key factor, while age, employment, and healthcare use show minimal influence. Cancer is linked to chronic stress, poorer perceived health, and mental health challenges. Risk is higher among older adults and those who keep house. Poor self-rated health, high BMI, low fruit and vegetable intake, and psychological distress increase risk, but healthcare use is not a strong predictor. **Conclusions**: While different chronic illnesses present distinct challenges to health behaviors and psychological well-being, they also share common features-such as increased stress and lifestyle disruptions-underscoring the importance of both tailored and cross-cutting interventions to effectively support individuals across conditions.

## 1. Introduction

Chronic conditions, also known as noncommunicable diseases (NCDs), are long-term health disorders that persist for a year or more and often require ongoing medical care or lifestyle adjustments [1]. These conditions, such as diabetes, cardiovascular diseases, chronic respiratory illnesses, and arthritis, are characterized by their prolonged duration, complex etiology, and significant impact on individuals’ quality of life [2]. Unlike acute illnesses, chronic conditions are rarely cured completely; instead, they are managed through multidisciplinary approaches, including pharmacological treatments, behavioral interventions, and self-management strategies [3]. They disproportionately affect aging populations and are linked to modifiable risk factors like poor diet, physical inactivity, and tobacco use, as well as non-modifiable factors such as genetic predisposition [4]. Chronic conditions not only strain healthcare systems but also contribute to economic burdens due to increased healthcare costs and reduced productivity [1]. Their effective management requires integrated care models that prioritize patient education, preventive measures, and equitable access to resources [3].

Hypertension, diabetes, and cancer are among the most prevalent chronic conditions globally, though their ranking varies by region and population. Hypertension is the most common chronic condition worldwide, affecting an estimated 1.3 billion adults [5]. Diabetes, particularly type 2, impacts over 537 million adults globally, with its prevalence rising due to obesity and sedentary lifestyles [6,7]. Cancer, while less prevalent than hypertension or diabetes, remains a leading cause of mortality, with 19.3 million new cases diagnosed annually [8]. In the U.S., hypertension affects nearly 48% of adults, diabetes about 13.6%, and cancer impacts one in three individuals over their lifetime [2,9].

Hypertension, diabetes, and cancer are conditions with distinct etiologies. Hypertension (high blood pressure) is characterized by persistently elevated blood force against arterial walls, often asymptomatic but a major risk factor for cardiovascular diseases, strokes, and kidney damage [10]. Diabetes, primarily type 2, involves impaired insulin production or utilization, leading to chronic hyperglycemia that damages blood vessels, nerves, and organs over time, increasing risks of heart disease, kidney failure, and blindness [7,11]. Cancer encompasses a group of diseases marked by uncontrolled cell growth and the invasion of surrounding tissues, driven by genetic mutations, environmental exposures (e.g., tobacco, UV radiation), and lifestyle factors; it disrupts normal bodily functions and is a leading cause of mortality worldwide [8].

Hypertension, diabetes, and cancer, while distinct in their biological mechanisms, share modifiable risk factors rooted in health behaviors, making their prevention and management critically dependent on lifestyle interventions. Poor diet (e.g., excessive sodium, sugar, or processed foods) contributes to obesity, insulin resistance, and inflammation, exacerbating all three conditions [9]. Physical inactivity directly impairs metabolic health, increasing hypertension and diabetes risk, while also elevating cancer susceptibility through mechanisms like chronic inflammation [12]. Smoking and excessive alcohol use are well-established carcinogens and drivers of vascular damage, worsening hypertension and diabetes outcomes [10]. Beyond these, health perception-how individuals view their own health-shapes engagement in preventive care; those underestimating risks are less likely to adopt healthy behaviors [13]. Poor sleep quality disrupts hormonal balance (e.g., cortisol and insulin), worsening weight gain and glycemic control [14]. Excess weight, particularly visceral fat, is a central risk factor, as obesity promotes insulin resistance, hypertension, and hormone-related cancers (e.g., breast, colon) [15].

Hypertension, diabetes, and cancer impose significant psychological burdens that exacerbate their physical toll. Individuals with hypertension often experience chronic stress and anxiety due to the silent, asymptomatic nature of the condition and fears of cardiovascular complications, which can lead to emotional distress and reduced quality of life [16]. Diabetes is closely linked to diabetes distress, a condition marked by feelings of frustration, burnout, and anxiety over relentless self-management tasks (e.g., glucose monitoring, dietary restrictions) and the threat of hypoglycemia or long-term complications like neuropathy [17]. Cancer diagnosis and treatment frequently trigger depression, fear of recurrence, and body image issues, with survivors reporting persistent psychological trauma even post-remission [18].

Chronic stress from these conditions can dysregulate the hypothalamic–pituitary–adrenal (HPA) axis, worsening inflammation and glycemic control and undermining immune function in cancer patients [19]. These psychological impacts not only diminish mental well-being but also reduce adherence to treatment, creating a cyclical relationship where poor mental health worsens physical outcomes. Integrated care models that address both psychological and physiological needs are critical to mitigating these effects.

Adults aged 50 and older are significant users of healthcare services, accounting for a disproportionately high share of medical visits, hospital stays, and prescription drug use compared to younger age groups [20]. As people age, the likelihood of living with one or more chronic conditions rises, which in turn drives greater demand for ongoing medical care, specialist consultations, and medication management [21]. Older adults typically visit physicians more frequently—often for chronic disease management or follow-up care after procedures—and are more likely than younger adults to require hospital admissions and long-term care [21,22]. Despite this high level of healthcare utilization, people over 50 often encounter barriers to accessing care. These include difficulties securing appointments, navigating digital health systems, and coping with long waiting times—challenges that are especially pronounced for those with complex health needs or those living in rural areas [23,24]. Furthermore, preventive healthcare services remain underused in this population, even though regular screenings and health education could help delay the progression of chronic illnesses [22]. Social support, financial resources, and geographic location also play a crucial role in shaping how and when older adults use healthcare services. Income inequality and limited mobility pose additional challenges for many in this age group, further influencing their access to care [24,25]. Overall, the healthcare utilization patterns of adults over 50 reflect both their higher medical needs and the systemic barriers they face in accessing timely and appropriate care.

Hypertension, diabetes, and cancer were selected for this study due to their highly significant health impact and distinct behavioral and psychological challenges. Together, they represent a broad spectrum of chronic illness experiences—from the often silent nature of hypertension to the self-management demands of diabetes and the life-altering effects of cancer. Despite differing biologically, these conditions share modifiable risk factors like poor diet, inactivity, and smoking, making them suitable for studying how lifestyle influences health outcomes. Their unique psychosocial burdens also allow for meaningful comparisons in mental well-being and coping across chronic illnesses.

Existing research on chronic conditions often overlooks the distinct psychological and behavioral profiles of hypertension, diabetes, and cancer, treating them as a homogeneous group rather than addressing their unique challenges. Socioeconomic and cultural determinants, such as financial instability or systemic discrimination, remain underexplored in how they intersect with disease management. There is also a lack of tailored interventions that account for condition-specific behavioral complexities, such as binge drinking patterns in hypertension or financial counseling needs in diabetes, despite their shared modifiable risk factors like poor diet and physical inactivity. The role of health perception and self-efficacy in shaping health behaviors is another understudied area, particularly how negative self-rated health perpetuates unhealthy practices even among those with moderate optimism. While negative psychological outcomes like depression are well-documented, positive psychological constructs such as resilience, generativity, and life satisfaction receive minimal attention despite their potential to improve coping and treatment adherence. Thus, this study sought to examine the various ways in which different long-term health conditions influence health-related behaviors and mental well-being, particularly focusing on recognizing and tackling the specific difficulties experienced by those living with high blood pressure, diabetes, and cancer. Accordingly, it is hypothesized that individuals with different chronic illnesses-namely hypertension, diabetes, and cancer-will demonstrate significantly distinct health behaviors, including medication adherence, physical activity, and dietary habits (Hypothesis 1). Additionally, it is expected that psychological well-being-reflected in levels of anxiety, depression, and perceived stress-will differ significantly across these diagnostic groups, given the unique psychosocial challenges associated with each condition (Hypothesis 2).

## 2. Materials and Methods

### 2.1. Ethics

This study was conducted in full accordance with established ethical standards for research involving human participants. Prior to commencement, the research protocol received formal approval from the Conselho Científico da Faculdade de Filosofia e Ciências Sociais da Universidade Católica Portuguesa, ensuring compliance with both institutional and national guidelines for ethical oversight. The study adhered to the principles of the Declaration of Helsinki, which provides a comprehensive ethical framework for medical and scientific research with human subjects [26]. The core principles of the Declaration include respect for participant autonomy, dignity, and rights, the necessity of informed consent, and the prioritization of participant welfare above scientific or societal interests. The Declaration also mandates the independent ethics committee review of research protocols and the strict protection of participant confidentiality and privacy throughout the study [26]. Data analysis for this study was conducted using a public database available upon request. Utilizing secondary data from a public repository promotes transparency and reproducibility while minimizing risks to individual privacy and well-being [27]. All the data were fully anonymized, and no attempts were made to identify or contact individual subjects. Access to the database was obtained in accordance with the repository’s terms of use and ethical guidelines, and all procedures related to data handling and analysis strictly adhered to applicable data protection standards [27]. By implementing these measures, the study upheld high ethical standards, safeguarded participants’ rights and confidentiality, and contributed responsibly to the advancement of scientific knowledge.

### 2.2. Instrument

The Americans’ Changing Lives (ACL) study is a nationally representative, longitudinal survey that examines how socioeconomic, psychological, and health factors evolve across adulthood. Initiated in 1986 by the National Institute on Aging (NIA) [28], its primary aim is to understand how social, psychological, and economic influences affect health and well-being throughout adult life, with a particular emphasis on disparities between Black and White Americans during mid-life and older age. Data have been collected across six waves, from the study’s launch in 1986 through to the most recent wave in 2019–2021. The initial sample included 3617 adults aged 25 and older, with deliberate oversampling of Black Americans and individuals aged 60 and above. In each subsequent wave, surviving participants were re-interviewed to monitor changes over time. The ACL study gathers extensive information on physical and mental health, health behaviors (such as smoking, alcohol use, and physical activity), psychological well-being (including depression and stress), social relationships and support networks, major life events, coping strategies, socioeconomic status, work history, and demographic details like age, race, and education.

#### 2.2.1. Sociodemographic Variables

The sociodemographic variables included age (continuous), gender (categorical; male and female), marital status (categorical; married, widowed, divorced, never married), and employment status (categorical; retired, working, unemployed, family caregiver).

#### 2.2.2. Clinical Variables

The clinical variables included chronic conditions (hypertension, diabetes, and cancer (all binary; yes and no)) and healthcare utilization (hospital admissions, emergency room visits, health professional visits, and mental health visits (all continuous variables)).

#### 2.2.3. Health Behavior Variables

The health behavior variables included self-rated health (ordinal scale; very good, good, and poor); dietary habits: fruit/vegetable intake (ordinal; 1–2 servings/day, 3–4 servings/day, 5+ servings/day); sleep patterns: sleep duration (continuous) and sleep disturbance (ordinal scales for trouble falling/staying asleep (e.g., rarely/never, almost daily)); substance use (smoking: binary (ever smoked and current smoker), with metrics for frequency (days/week: continuous) and intensity (cigarettes/day: continuous); alcohol use (binary (drinker: yes or no)), with frequency (days/month: continuous) and quantity (drinks/occasion: continuous); physical health metrics: weight (continuous) and Body Mass Index (BMI) (continuous, obese [BMI ≥ 30]); and physical activity (standardized index, negative values indicate below-average activity).

#### 2.2.4. Psychological Variables

The Positive Psychological Indicators (standardized indices) included the following: life satisfaction (1–7 scale; lower scores indicate higher satisfaction); optimism (scale; lower scores indicate higher satisfaction); self-esteem (negative values reflect lower self-esteem); meaning and generativity (higher values indicate greater purpose); and self-efficacy (negative values reflect lower confidence).

The Negative Psychological Indicators (standardized indices) included the following: loneliness (higher values indicate greater loneliness); the Chronic Parental Stress Index (scale; lower scores indicate higher satisfaction); depressive symptoms (CESD-11 scale) (negative values indicate fewer symptoms); cognitive impairment (higher values indicate greater impairment); anger-out (higher values indicate outward anger expression); cynical hostility (higher values indicate mistrust); hopelessness (higher values indicate pessimism); everyday discrimination (higher values indicate perceived bias); vigilance (higher values indicate hyper-alertness); and chronic financial stress (negative values indicate lower stress). All the indices were calculated by the original researchers of the Americans’ Changing Lives (ACL) study [28] and were included in the publicly available dataset.

### 2.3. Statistical Analysis

Descriptive statistics were first calculated to provide an overview of the dataset. For continuous variables, measures such as the mean were used to summarize the central tendency, while the standard deviation quantified variability. For categorical variables, frequencies and percentages were reported to describe the distribution of responses.

To examine associations between categorical variables, chi-squared tests of independence were performed. The null hypothesis for these tests stated that there was no association between the variables under consideration. The assumptions of the chi-squared tests, including adequate expected cell counts and the independence of observations, were checked prior to analysis. To assess the strength of any observed associations, the Phi coefficient was calculated for 2 × 2 contingency tables. The Phi coefficient is derived from the chi-squared statistic and provides a measure of effect size, with values of 0.1, 0.3, and 0.5 generally interpreted as small, medium, and large effects, respectively.

To compare means between two independent groups, independent samples t-tests were conducted. Prior to performing the *t*-tests, assumptions of normality and homogeneity of variances were evaluated using the Shapiro–Wilk and Levene tests, respectively. Alongside the *t*-test results, Cohen’s d was calculated to quantify the magnitude of the difference between group means. Cohen’s d is interpreted as showing small (0.2), medium (0.5), or large (0.8) effect sizes. Bonferroni-adjusted *p*-values were calculated to control for Type I errors across all comparisons (corrected alpha = 0.01). Comparisons with *p*-values below this threshold remained statistically significant, indicating robust differences even after adjustment. All other comparisons had *p*-values greater than 0.01 and were not considered significant following Bonferroni correction.

Logistic regression was used to analyze the relationship between all the independent variables (sociodemographic, clinical, health behavior, and psychological variables), and as dependent variables, hypertension, diabetes, and cancer. In logistic regression, regression coefficients (B) represent the change in log odds for each unit increase in a predictor, while odds ratios (Exp(B)) express this effect in more interpretable terms. Confidence intervals, typically at 95%, show the precision of these estimates, and if they include 1, the predictor may not be significant. *p*-values test the significance of each coefficient, with values below 0.05 indicating statistical significance. The Wald statistic further supports the relevance of each predictor. Model fit was evaluated using the −2 log likelihood, chi-square test, and pseudo-R-squared values like Nagelkerke’s R^2^. A classification table provided accuracy metrics, and the Hosmer–Lemeshow test checked the model’s goodness-of-fit, where a non-significant result indicated a good fit.

To assess the robustness of the results, sensitivity analyses were conducted by excluding cases with standardized residuals above ±2 and Cook’s Distance greater than 1.

All statistical analyses were performed using SPSS, version 30. The results were reported with corresponding test statistics, degrees of freedom, *p*-values, and effect sizes.

## 3. Results

### 3.1. Sociodemographics

Data were collected in 2021 (Wave 6) [29], and all participants were American. Information on participants’ ethnicity was not available for Wave 6 [29]. Among the valid responses (767), 40% of respondents identified as male (307 individuals) and 60% as female (460 individuals). Respondents had an average age of 70.09 years (median: 68), indicating a predominantly older population. The standard deviation of 8.8 years and range from 58 to 99 years show moderate variability.

A majority of valid respondents (53.2%) were married, followed by being widowed (18.1%), divorced (17.7%), or never married (9.4%). Over half of the valid respondents (56.2%) were retired, while 28.6% reported that they were currently working. Other categories, such as being unemployed (1.3%) or family caregivers (0.4%), were minimal. The high retirement rate further supports the older age profile of the sample.

### 3.2. Clinical Variables

Among the clinical variables examined, the most commonly reported condition was hypertension. Fewer individuals had a history of diabetes or cancer. The majority of participants did not report having chronic illnesses such as cancer or diabetes (Table 1).

### 3.3. Clinical Services

Table 2 provides a descriptive overview of healthcare utilization across various services, reflecting both physical and mental health needs. While engagement with general health professionals appears high, other services such as emergency room and hospital admissions were less frequently reported. Mental health services showed a moderate level of use, suggesting a measurable reliance on psychological support within the sample.

### 3.4. Health Behavior Variables

This study data included 19 health-related behaviors across a population survey, focusing on lifestyle factors such as diet, sleep, substance use, and physical health metrics. Self-rated health was generally positive, with most respondents describing their health as “very good” or “good,” while a small minority reported “poor” health. Dietary habits showed limited adherence to recommended fruit and vegetable intake, with fewer than a fifth consuming five or more servings daily. Sleep patterns indicated adequate average sleep duration, though a subset reported difficulties falling asleep or staying asleep, with some experiencing frequent disruptions.

Smoking behaviors revealed that over half of respondents had smoked at some point, though current smokers represented a smaller subgroup. Most smokers reported daily use, with attempts to quit being common but limited use of cessation aids. Alcohol consumption was widespread, with drinkers showing variability in frequency, from abstinence to daily use, though the typical intake per occasion was moderate. Weight and BMI highlighted concerns around obesity, with a notable proportion classified as obese. Sleep duration, smoking metrics, and alcohol use also included unusual or extreme responses that warrant further scrutiny.

Concerning physical activity, negative scores reflect activity levels relative to the sample mean, not absolute inactivity. Regarding distribution, most responses cluster around the mean (−0.12), with moderate variability (SD: 1.03) (Table 3).

### 3.5. Positive Psychological Variables

The Positive Psychological Indicators of this study are life satisfaction, optimism, self-esteem, meaning and generativity, and self-efficacy. Concerning life satisfaction, the low mean indicates a generally high level of life satisfaction among participants. The median supports this, showing consistency across the group. Regarding the Optimism Index, the mean of 3.10 shows that optimism is moderate. Participants generally hold a slightly positive outlook on the future, which is encouraging, although there is room for enhancement. The mean and median of the Self-Esteem Index are both below zero, reflecting low self-esteem relative to normative expectations. Concerning the Meaning and Generativity Index, this index shows the most positive result, with both its mean and median well above 1. Finally, the scores of the Self-Efficacy Index are the lowest of the group, with both the mean and median significantly below zero (Table 4).

### 3.6. Negative Psychological Variables

In Table 5, we find the mean, median, and standard deviation for each of the negative psychological variables. The Loneliness Index, with a mean of 3.10, stands out as the highest among all the measured variables. The Chronic Parental Stress Index, which has a slightly negative mean of −0.19 (standardized), suggests a mild reduction or below-average level of parental stress, though this can vary across individuals. The Cognitive Impairment Index shows a mean of 0.55, pointing to a moderate presence of self-reported or perceived cognitive difficulties. Depressive symptoms, measured through the CESD-11 scale, have a mean of −0.21. This negative value implies fewer depressive symptoms on average. The Anger-Out Index, with a mean of 1.37, reflects a moderate tendency to express anger outwardly. The Cynical Hostility Index records a relatively high mean of 2.45.

Hopelessness, with a mean score of 1.79, appears moderately high. Similarly, the Everyday Discrimination Index has a mean of 1.66, pointing to a tangible perception of unfair treatment. The Vigilance Index presents a high mean of 2.60, indicating a heightened state of alertness or sensitivity to potential threats. Lastly, the Chronic Financial Stress Index, with a mean of −0.40, reveals that, on average, participants do not report severe financial stress, or that their stress levels are below the normative baseline for such concerns (Table 5).

### 3.7. Relationship Between Chronic Illness and Positive and Negative Psychological Variables

#### 3.7.1. Hypertension

Concerning the variable “hypertension”, the data shows the distribution of life satisfaction levels among individuals who have been told by a healthcare provider that they have hypertension versus those who have not. In fact, 19% of hypertensive individuals are completely satisfied with their lives compared to 16.1% of non-hypertensive individuals. Similarly, 47.8% of hypertensive individuals are very satisfied, 27.8% are somewhat satisfied, 3.6% are not very satisfied, and 1.8% are not at all satisfied (χ^2^(4) = 5.633, *p* = 0.228, ϕ = 0.09). The Optimism Index shows that higher optimism scores are generally less frequent among hypertensive individuals (χ^2^(9) = 13.396, *p* = 0.145, ϕ = 0.138). The Self-Esteem Index shows varied levels among hypertensive and non-hypertensive individuals, with a notable concentration of scores around the middle range (χ^2^(41) = 40.957, *p* = 0.473, ϕ = 0.242). The Self-Efficacy Index measures individuals’ belief in their ability to succeed in specific situations. The data indicates a wide range of self-efficacy scores among hypertensive individuals (χ^2^(228) = 237.275, *p* = 0.323, ϕ = 0.582).

The Loneliness Index suggests a higher prevalence of loneliness among hypertensive individuals compared to non-hypertensive individuals (χ^2^(9) = 5.513, *p* = 0.788, ϕ = 0.089). The Chronic Parental Stress Index measures the stress levels related to parenting. Hypertensive individuals show varied stress levels, with some higher stress scores (χ^2^(54) = 62.395, *p* = 0.202, ϕ = 0.341). The Cognitive Impairment Index indicates a higher percentage of cognitive impairment among hypertensive individuals (χ^2^(5) = 18.390, *p* = 0.002, ϕ = 0.162). The CESD-11 Depressive Symptoms Index is higher in participants with hypertension. The chi-square test for this relationship has a Pearson value of (χ^2^(327) = 332.480, *p* = 0.406, ϕ = 0.689). The Anger-Out Index measures outward expressions of anger, with hypertensive individuals showing higher scores (χ^2^(9) = 6.566, *p* = 0.682, ϕ = 0.097). The Cook–Medley Cynical Hostility Index measures cynical hostility, with hypertensive individuals showing higher levels (χ^2^(15) = 15.528, *p* = 0.414, ϕ = 0.149). The Hopelessness Index suggests a higher prevalence of hopelessness among hypertensive individuals (χ^2^(9) = 14.717, *p* = 0.099, ϕ = 0.145). The Everyday Discrimination Index measures perceived discrimination, with hypertensive individuals reporting higher levels (χ^2^(17) = 15.825, *p* = 0.536, ϕ = 0.15). The Vigilance Index measures vigilance, with hypertensive individuals showing higher scores (χ^2^(32) = 27.810, *p* = 0.679, ϕ = 0.199). The Number of Recent Life Events Index includes the number of recent life events experienced by individuals, showing a higher frequency among hypertensive individuals (χ^2^(5) = 4.171, *p* = 0.525, ϕ = 0.076). The Chronic Financial Stress Index measures financial stress, with hypertensive individuals showing higher levels (χ^2^(25) = 34.061, *p* = 0.107, ϕ = 0.221).

However, none of the differences between groups are statistically significant, except for the Cognitive Impairment Index, which reveals a significantly higher prevalence of cognitive impairment among hypertensive individuals compared to their non-hypertensive counterparts.

#### 3.7.2. Diabetes

The Life Satisfaction Index suggests no significant differences in distribution between diabetic and non-diabetic individuals (χ^2^(4) = 5.948, *p* = 0.203, ϕ = 0.094). Similarly, the Optimism Index shows no significant difference (χ^2^(9) = 16.286, *p* = 0.061, ϕ = 0.156). The Self-Esteem Index also indicates no significant differences between groups (χ^2^(40) = 49.796, *p* = 0.138, ϕ = 0.273). The Self-Efficacy Index suggests a moderate effect size, though the chi-square test result is not significant (χ^2^(217) = 251.746, *p* = 0.053, ϕ = 0.613). The Loneliness Index reveals no significant differences between diabetic and non-diabetic individuals (χ^2^(9) = 13.109, *p* = 0.158, ϕ = 0.140). The Chronic Parental Stress Index similarly shows no significant differences (χ^2^(51) = 48.859, *p* = 0.559, ϕ = 0.309). The Cognitive Impairment Index indicates very weak differences, which are not statistically significant (χ^2^(5) = 4.469, *p* = 0.484, ϕ = 0.082). In contrast, the Depressive Symptoms Index reveals a significantly higher prevalence among diabetic individuals (χ^2^(314) = 387.988, *p* = 0.003, ϕ = 0.762). The Anger-Out Index shows no significant differences (χ^2^(8) = 3.329, *p* = 0.912, ϕ = 0.071), while the Cynical Hostility Index also reveals no significant differences between groups (χ^2^(15) = 16.249, *p* = 0.366, ϕ = 0.156). The Hopelessness Index suggests significantly higher levels among diabetic individuals (χ^2^(9) = 23.943, *p* = 0.004, ϕ = 0.189). The Everyday Discrimination Index shows no significant differences (χ^2^(17) = 20.679, *p* = 0.241, ϕ = 0.176). The Vigilance Index shows no statistically significant differences between groups (χ^2^(32) = 43.661, *p* = 0.082, ϕ = 0.256). The Number of Recent Life Events Index reveals no significant differences (χ^2^(5) = 8.223, *p* = 0.144, ϕ = 0.110). The Chronic Financial Stress Index reveals significantly higher levels among diabetic individuals (χ^2^(26) = 48.310, *p* = 0.005, ϕ = 0.269). However, only the Depressive Symptoms, Hopelessness, and Chronic Financial Stress indices reveal statistically significant differences between diabetic and non-diabetic individuals.

#### 3.7.3. Cancer

The Life Satisfaction Index reveals no significant differences between individuals with and without a cancer diagnosis (χ^2^(4) = 3.074, *p* = 0.546, ϕ = 0.068). Similarly, the Optimism Index shows no significant difference (χ^2^(9) = 2.480, *p* = 0.981, ϕ = 0.061). The Self-Esteem Index indicates no significant differences between groups (χ^2^(40) = 51.356, *p* = 0.108, ϕ = 0.279). The Self-Efficacy Index also shows no statistically significant difference (χ^2^(214) = 215.105, *p* = 0.466), although the Phi coefficient (ϕ = 0.571) points to a moderate relationship. The Loneliness Index reveals no significant difference between individuals with and without cancer (χ^2^(9) = 5.585, *p* = 0.781, ϕ = 0.092). In contrast, the Chronic Parental Stress Index shows a statistically significant difference, with higher levels observed in individuals with cancer (χ^2^(50) = 71.351, *p* = 0.025, ϕ = 0.377). The Cognitive Impairment Index presents no significant difference (χ^2^(5) = 5.764, *p* = 0.330, ϕ = 0.094). The Depressive Symptoms Index does not show a statistically significant difference (χ^2^(304) = 308.311, *p* = 0.420), although the Phi coefficient (ϕ = 0.684) suggests a potentially strong pattern. The Anger-Out Index (χ^2^(8) = 8.796, *p* = 0.360, ϕ = 0.116), the Cynical Hostility Index (χ^2^(15) = 8.511, *p* = 0.902, ϕ = 0.114), and the Hopelessness Index (χ^2^(9) = 13.712, *p* = 0.133, ϕ = 0.144) all indicate weak or very weak differences. The Everyday Discrimination Index does not show significant differences (χ^2^(17) = 20.166, *p* = 0.266, ϕ = 0.175), and the Vigilance Index similarly lacks significance (χ^2^(31) = 32.128, *p* = 0.411, ϕ = 0.221), though the latter suggests a moderate relationship. The Number of Recent Life Events Index reveals no significant differences (χ^2^(5) = 8.147, *p* = 0.148, ϕ = 0.110). Lastly, the Chronic Financial Stress Index shows no significant differences between groups (χ^2^(25) = 19.265, *p* = 0.784, ϕ = 0.171). Among the psychological indices analyzed, only the Chronic Parental Stress Index shows statistically significant differences between individuals with and without a cancer diagnosis.

### 3.8. Relationship Between Chronic Illness and Health Behavior Variables

#### 3.8.1. Hypertension

The analysis of health behaviors and outcomes revealed several significant differences between participants diagnosed with hypertension and those without hypertension. Significant differences (*p* < 0.05) were found related to self-rated health, problems falling asleep, problems waking up and getting back to sleep, weight, alcohol consumption, days drinking alcohol per month, number of drinks per day, physical activity, and Body Mass Index (BMI).

Participants with hypertension rated their health significantly worse (mean = 2.77) compared to those without hypertension (mean = 2.28). This difference is highly significant (*p* < 0.001), with a moderate effect size (Cohen’s d = 0.506), suggesting that hypertension has a substantial negative impact on perceived health status (Table 6).

Participants with hypertension reported more problems falling asleep (mean = 1.78) than those without hypertension (mean = 1.59). This difference is statistically significant (*p* = 0.004), with a small effect size (Cohen’s *d* = 0.222), indicating potential sleep disturbances associated with hypertension. Also, participants with hypertension experienced more difficulties waking up and getting back to sleep (mean = 1.97) compared to those without hypertension (mean = 1.82). This difference is statistically significant (*p* = 0.020), with a small effect size (Cohen’s d = 0.178) (Table 6).

Participants with hypertension reported higher frequencies of drinking alcoholic beverages (mean = 2.67) compared to those without hypertension (mean = 2.36). This difference is statistically significant (*p* = 0.036), with a small effect size (Cohen’s *d* = 0.158). In addition, participants with hypertension drank on fewer days in the past month (mean = 6.79 days) compared to those without hypertension (mean = 8.94 days). This difference is statistically significant (*p* = 0.019), with a small effect size (Cohen’s *d* = −0.225), revealing complex alcohol consumption patterns. Participants with hypertension consumed more drinks per day (mean = 2.00) compared to those without hypertension (mean = 1.63). This difference is statistically significant (*p* = 0.037), with a small effect size (Cohen’s *d* = 0.223) (Table 6).

Participants with hypertension weighed more on average (mean = 187.17 units) compared to those without hypertension (mean = 175.91 units). This difference is statistically significant (*p* < 0.001), with a small-to-medium effect size (Cohen’s *d* = 0.254), consistent with established links between weight and hypertension. Also, participants with hypertension had a higher BMI (mean = 29.49) compared to those without hypertension (mean = 27.65). This difference is highly significant (*p* < 0.001), with a small-to-medium effect size (Cohen’s *d* = 0.296), reinforcing the relationship between higher BMI and hypertension risk. Finally, participants with hypertension had significantly lower physical activity levels (mean = −0.28) compared to those without hypertension (mean = 0.09). This difference is highly significant (*p* < 0.001), with a medium effect size (Cohen’s *d* = −0.370), highlighting an important area for potential intervention (Table 6).

However, there are some non-significant differences. In fact, the following health behaviors did not differ significantly between those with and without hypertension: fruit and vegetable intake, total sleep time (hours and minutes), smoking status and behaviors (ever smoked, smoked more than 100 cigarettes, current smoking status, cigarettes per day, quit attempts, and use of cessation products or services).

#### 3.8.2. Diabetes

The analysis of health behaviors and outcomes revealed several significant differences between participants diagnosed with diabetes and those without diabetes. Significant differences (*p* < 0.05) were found related to self-rated health, problems falling asleep, weight, alcohol consumption, physical activity, and Body Mass Index (BMI). Participants with diabetes rated their health significantly worse (mean = 3.10) compared to those without diabetes (mean = 2.41). This difference is highly significant (*p* < 0.001), suggesting that diabetes has a substantial negative impact on perceived health status. Also, participants with diabetes reported more problems falling asleep (mean = 1.83) than those without diabetes (mean = 1.65). This difference is statistically significant (*p* = 0.029), indicating potential sleep disturbances associated with diabetes. Participants with diabetes weighed more on average (mean = 190.35 units) compared to those without diabetes (mean = 179.86 units). This difference is statistically significant (*p* = 0.012), consistent with established links between weight and type 2 diabetes. In addition, participants with diabetes reported a higher frequency of drinking alcoholic beverages (mean = 3.00) compared to those without diabetes (mean = 2.38). However, those with diabetes drank on fewer days in the past month (mean = 4.89 days) compared to those without diabetes (mean = 8.36 days). Both differences are statistically significant (*p* < 0.001 and *p* = 0.005, respectively), revealing complex alcohol consumption patterns in these populations. Concerning physical activity, participants with diabetes had significantly lower physical activity levels (mean = −0.43) compared to those without diabetes (mean = −0.03). This difference is highly significant (*p* < 0.001), highlighting an important area for potential intervention. At last, participants with diabetes had a higher BMI (mean = 29.56) compared to those without diabetes (mean = 28.42). This difference is marginally significant (*p* = 0.051 for the standard test and *p* = 0.049 with unequal variances assumed), reinforcing the relationship between higher BMI and diabetes risk (Table 7).

However, there are some non-significant differences. In fact, the following health behaviors did not differ significantly between those with and without diabetes: fruit and vegetable consumption, total sleep time (hours and minutes), problems waking up and getting back to sleep, smoking status and behaviors (ever smoked, currently smoking, cigarettes per day, quit attempts), and number of drinks consumed on days when drinking.

#### 3.8.3. Cancer

Individuals with cancer rate their health slightly lower (mean = 2.69) compared to those without cancer (mean = 2.49). This difference is statistically significant (*p* = 0.045), suggesting that having cancer negatively impacts self-perceived health.

Both groups consume similar amounts of fruits and vegetables daily (mean = 2.31 servings). There is no significant difference between the groups (*p* = 0.926), indicating that cancer status does not affect fruit and vegetable consumption. The average sleep duration in hours is almost identical for both groups (yes: 7.29 h; no: 7.31 h). There is no significant difference (*p* = 0.879), suggesting that cancer status does not impact the amount of sleep. Problems falling asleep and waking up are slightly more frequent in individuals with cancer, but the differences are not statistically significant (*p* > 0.05). This suggests that while sleep issues may be more common in those with cancer, the difference is not substantial. The average weight is similar between the two groups (yes: 181.01 kg; no: 182.14 kg), with no significant difference (*p* = 0.793). This indicates that cancer status does not significantly affect weight.

The proportion of those who have ever smoked is similar in both groups (yes: mean = 2.88; no: mean = 2.84), with no significant difference (*p* = 0.851). This suggests that smoking history is comparable regardless of cancer status. Current smoking rates are also similar (yes: mean = 4.47; no: mean = 4.40), with no significant difference (*p* = 0.730). This indicates that cancer status does not significantly influence current smoking behavior. The number of days smoked per week and the number of cigarettes smoked per day are slightly higher in individuals with cancer, but the differences are not significant (*p* > 0.05). This suggests that smoking habits are relatively consistent across both groups. The mean number of quitting attempts is similar (yes: mean = 1.75; no: mean = 1.83), with no significant difference (*p* = 0.741). This indicates that both groups have made similar efforts to quit smoking.

The average frequency of alcohol consumption is similar between the two groups (yes: mean = 2.52; no: mean = 2.49), with no significant difference (*p* = 0.870). This suggests that cancer status does not significantly affect alcohol consumption. The number of days that alcohol is consumed per month and the number of drinks per drinking day are similar between the groups, with no significant differences (*p* > 0.05). This indicates that drinking habits are consistent regardless of cancer status.

The average BMI is similar among the two groups (yes: mean = 28.50; no: mean = 28.62), with no significant difference (*p* = 0.835). This suggests that cancer status does not significantly affect BMI. The Physical Activity Index is slightly lower in individuals with cancer (yes: mean = −0.20) compared to those without (no: mean = −0.07), but the difference is not significant (*p* = 0.196). This indicates that physical activity levels are relatively similar across both groups.

### 3.9. Logistic Regressions

Logistic regressions were employed to examine how age, gender, marital status, and employment status are associated with the likelihood of having hypertension, diabetes, or cancer. After adjusting for covariates, age (OR = 1.037, 95% CI [1.013, 1.061]), widowed marital status (OR = 2.40, 95% CI [1.05, 5.48]), and retirement (OR = 7.6, 95% CI [1.66, 34.8]) emerged as significant hypertension predictors. Holding all other variables constant, the odds of hypertension increased by 3.7% for each additional year of age. Widowed individuals had 2.4 times higher odds of hypertension compared to their married counterparts, adjusting for other factors. Retired individuals showed 7.6 times higher odds of hypertension than those currently working. Female sex was associated with 10% higher odds of hypertension compared to males, though this was not statistically significant (OR = 1.10, 95% CI [0.788, 1.534]). Caution is warranted due to the quasi-separation in employment categories and marginal model fit (Nagelkerke R = 0.109). The Hosmer–Lemeshow test (*p* = 0.735) indicates adequate fit.

After adjusting for covariates, female sex (OR = 0.52, 95% CI [0.33–0.81]) and separated marital status (OR = 7.25, 95% CI [1.40–37.5]) emerged as significant diabetes predictors. Holding other variables constant, females had 48% lower odds of diabetes compared to males; separated individuals showed 7.25 times higher odds of diabetes than married counterparts. Widowed marital status showed borderline significance (OR = 1.73, 95% CI [0.98–3.06], *p* = 0.059). Age and employment status categories exhibited non-significant effects or extreme coefficients due to data limitations. This logistic regression model demonstrated adequate goodness-of-fit but limited explanatory power (Nagelkerke R^2^ = 0.105), indicating that the predictors explain approximately 10.5% of the variance in diabetes diagnosis. The Hosmer–Lemeshow test yielded a non-significant p-value (*p* = 0.260), supporting the model’s ability to reliably distinguish between individuals with and without diabetes.

After adjusting for covariates, age (OR = 0.963, 95% CI [0.938, 0.989]) and “Keeping House” employment status (OR = 2.687, 95% CI [1.039, 6.950]) emerged as significant cancer predictors. Each additional year increased cancer odds by 3.7% (OR = 0.963, 95% [0.938, 0.989], *p* = 0.005). Employment status (keeping house) is associated with 2.7 times higher odds of cancer vs. working individuals. Model explains 6.4% of variance (Nagelkerke R^2^ = 0.064). The Hosmer–Lemeshow test for the cancer logistic regression model yielded a chi-square value of 10.596 with eight degrees of freedom and a significance (*p*) value of 0.226, indicating that there was no significant difference between the observed and expected values predicted by the model. In practical terms, this means the model demonstrates adequate goodness-of-fit.

A logistic regression analysis examining the relationship between healthcare utilization and hypertension diagnosis showed a statistically significant improvement over the null model (χ^2^ = 30.019, df = 4, *p* < 0.001). However, the explained variance was modest, with a Cox & Snell R^2^ of 0.042 and a Nagelkerke R^2^ of 0.056, indicating that the model accounted for only 5.6% of the variance in hypertension diagnosis. The classification accuracy increased from a baseline of 55.9% to 61.3%, representing a modest improvement. Among the predictors, hospital admissions in the past 12 months were significantly associated with higher odds of hypertension diagnosis (B = 0.066, OR = 1.068, *p* = 0.044), suggesting a 6.8% increase in odds per additional admission. Emergency room visits showed a significant negative association (B = −0.282, OR = 0.754, *p* < 0.001), with each visit reducing the odds of diagnosis by 24.6%. Similarly, visits to healthcare professionals such as doctors, nurse practitioners, and physician assistants were also negatively associated (B = −0.037, OR = 0.964, *p* = 0.005), with each visit linked to a 3.6% decrease in the odds of a hypertension diagnosis. Mental health visits had a positive but statistically non-significant association (B = 0.300, OR = 1.349, *p* = 0.248), indicating a 34.9% increase in odds that did not reach the threshold for significance.

A separate regression analysis of diabetes diagnosis found that the model did not significantly improve upon the null (χ^2^ = 7.754, df = 4, *p* = 0.101) and that the variance explained was very low (Cox & Snell R^2^ = 0.011; Nagelkerke R^2^ = 0.018). Although the Hosmer–Lemeshow test suggested acceptable model fit (χ^2^ = 14.678, df = 8, *p* = 0.066), the model’s predictive value was poor. None of the predictor variables reached statistical significance. Hospital admissions had a slightly negative, non-significant association (B = −0.022, OR = 0.979, *p* = 0.339), and emergency room visits showed a similar trend (B = −0.121, OR = 0.886, *p* = 0.133). Visits to healthcare professionals had minimal effect (B = −0.012, OR = 0.988, *p* = 0.194), and mental health visits were positively associated (B = 0.239, OR = 1.269, *p* = 0.415), but not significantly so. In the analysis of cancer diagnosis, the model also failed to show statistical significance (Omnibus *p* = 0.506) and explained less than 1% of the variance (Nagelkerke R^2^ = 0.008). While the Hosmer–Lemeshow test did not indicate poor fit (*p* = 0.276), none of the healthcare utilization variables were significantly associated with cancer diagnosis, further reflecting the model’s very limited predictive capability.

A logistic regression examining the relationship between hypertension and health behaviors found that self-rated health (*p* < 0.001), BMI (*p* < 0.001), and physical activity (*p* < 0.05) were significant predictors. Better self-rated health was associated with higher odds of hypertension (OR = 0.400), while higher BMI increased the odds (OR = 1.073) and greater physical activity slightly reduced them (OR = 0.985). The model had a good fit (Hosmer–Lemeshow χ^2^ = 17.737) and explained a modest portion of variance (Cox & Snell R^2^ = 0.091; Nagelkerke R^2^ = 0.121). For diabetes, self-rated health (*p* < 0.001) and BMI were significant, with odds ratios of 0.519 and 1.092, respectively, indicating that better self-rated health was linked to higher odds and higher BMI to increased odds. Physical activity showed a slight, non-significant positive association (OR = 1.007). The model fit was acceptable (Hosmer–Lemeshow χ^2^ = 17.635), and the variance explained was moderate (Cox & Snell R^2^ = 0.126; Nagelkerke R^2^ = 0.217). In the cancer model, self-rated health was a strong, significant predictor (*p* < 0.001, OR = 4.576), suggesting higher odds of diagnosis with worst self-perceived health. Fruit and vegetable consumption was associated with lower odds (OR = 0.489), and BMI showed a modest positive association (OR = 1.030). The model fit was acceptable (Hosmer–Lemeshow χ^2^ = 15.767), though the explained variance was low (Cox & Snell R^2^ = 0.030; Nagelkerke R^2^ = 0.050).

Logistic regression analyses were conducted to examine the relationship between psychological variables—life satisfaction, optimism, and depressive symptoms—and the likelihood of being diagnosed with hypertension, diabetes, or cancer. For hypertension, higher life satisfaction was significantly associated with a higher risk (odds ratio [OR] = 0.486, *p* < 0.001) and greater optimism was also linked to a higher risk (OR = 1.325). Higher levels of depressive symptoms were also associated with a higher likelihood of hypertension (OR = 0.341). The model demonstrated good fit according to the Hosmer and Lemeshow test (χ^2^ = 15.948), with a Cox & Snell R^2^ = 0.055 and a Nagelkerke R^2^ = 0.074.

In the case of diabetes, life satisfaction showed a strong positive association with risk (OR = 3.664, *p* < 0.001), indicating that individuals with the worst life satisfaction were more likely to have diabetes. Similarly, lower optimism was significantly associated with increased risk, and higher depressive symptoms were linked to higher risk. The model fit was acceptable (Hosmer and Lemeshow χ^2^ = 15.062), with a Cox & Snell R^2^ = 0.069 and a Nagelkerke R^2^ = 0.107.

For cancer diagnoses, higher life satisfaction was again associated with greater risk (OR = 4.020, *p* < 0.001), and lower optimism was similarly linked to an increased likelihood of diagnosis. Depressive symptoms were positively related to cancer risk. The model showed good fit (Hosmer and Lemeshow χ^2^ = 13.148), with a Cox & Snell R^2^ = 0.028 and a Nagelkerke R^2^ = 0.044.

Logistic regression results showed that age, marital status, and employment status were significant predictors of disease risk. Hypertension was associated with older age, being widowed, and retirement. Diabetes risk was higher among separated individuals and those with lower life satisfaction. Cancer risk increased with age and “keeping house.” Higher BMI, poorer self-rated health, and lower psychological well-being (life satisfaction, optimism, depressive symptoms) were linked to higher odds across all three diseases. While the models explained modest variance, they highlighted key sociodemographic, behavioral, and psychological risk factors.

The direction and significance of the key predictors remained largely consistent, suggesting that the findings were not unduly influenced by outliers.

## 4. Discussion

This study aimed to investigate chronic illnesses-specifically hypertension, diabetes, and cancer-affect health behaviors and psychological well-being. The research focused on identifying the unique challenges faced by individuals living with these conditions, with the broader goal of informing strategies to better meet their specific needs. To this end, data from Wave 6 [29] of the Americans’ Changing Lives study, comprising 767 participants, was analyzed. Variables relevant to both physical and mental health were selected for examination.

### 4.1. The Sample

The sample was predominantly composed of older adults, most of whom were female, married, and retired—demographic traits typical of senior populations. Chronic conditions such as hypertension, diabetes, and cancer disproportionately affect this age group and are closely linked to both modifiable and non-modifiable risk factors, including age and gender [1,4]. Nearly 95% of adults over 60 have at least one chronic illness, and around 80% live with multiple conditions [2]. Older women are especially impacted, experiencing higher rates of multimorbidity and diseases like hypertension and arthritis [9]. Marriage and retirement are also common in this group, reflecting life-stage norms. Together, these demographic patterns underscore the intersection of age, gender, and lifestyle in shaping the risk and management of noncommunicable diseases (NCDs).

### 4.2. Healthcare Utilization

The pattern of healthcare use observed—frequent visits to general health professionals, the moderate use of mental health services, and relatively low rates of emergency room visits and hospital admissions—mirrors established trends in adults over 50. Research consistently shows that general practitioners are the most commonly accessed providers in this age group [23,24,30]. This reflects the central role of primary care in managing chronic conditions, providing preventive care, and monitoring health in older adults [21,22]. In contrast, our results showed that emergencies and hospital services are used less often and typically for acute issues or complications from existing illnesses. Only about 20% of adults over 50 report an emergency visit annually [30]. These patterns highlight how most health concerns in this population are addressed in outpatient settings, emphasizing the need for coordinated, continuous care [1].

The moderate use of mental health services among older adults in our sample reflects broader trends. Despite the high prevalence of conditions like depression and anxiety in this population, access to care is often limited by stigma, a shortage of geriatric mental health providers, and the common under-recognition of mental illness by both patients and professionals [31,32]. Many older adults may mistake symptoms for normal aging, reducing help-seeking behavior [32], while ageism and a lack of specialized care further discourage treatment [33]. Nonetheless, the observed moderate engagement suggests a growing awareness of psychological needs, especially with the expansion of integrated care models [22,23].

Variability in healthcare use across the sample likely reflects differences in health status, social support, income, and access to services. Living alone, geographic isolation, and income disparities are known to affect service use, with some individuals requiring more intensive care due to complex needs or limited informal support [25,30]. Structural barriers such as transportation, appointment availability, and low digital literacy also continue to shape how older adults engage with care systems [34].

### 4.3. Clinical Variables

Hypertension was the most commonly reported condition among the participants. This finding aligns with the existing literature, which identifies hypertension as the most prevalent chronic disease worldwide, affecting an estimated 1.3 billion adults [5]. In the United States alone, nearly 48% of adults are diagnosed with hypertension, making it substantially more common than either diabetes or cancer [2,9]. Global data further emphasize its growing impact: the number of individuals with hypertension has doubled over the past three decades, now affecting approximately one in three adults [5]. Hypertension particularly burdens older adults—the majority demographic in this study’s sample—and is associated with both modifiable risk factors, such as diet, physical inactivity, and alcohol use, and non-modifiable factors, including age and genetics [1,4].

Although most respondents rated their health positively, many reported behaviors that elevate chronic disease risk, including poor diet, sleep difficulties, smoking, alcohol use, obesity concerns, and inconsistent physical activity. These findings align with broader evidence showing that modifiable behaviors—such as unhealthy eating, inactivity, substance use, and inadequate sleep—are key contributors to chronic conditions like hypertension, diabetes, and cancer [35]. Despite widespread awareness, adherence to health guidelines remains low among older adults, with persistent gaps between knowledge and behavior [36,37].

Common issues such as insufficient fruit and vegetable intake and poor sleep quality increase the risk of obesity and cardiometabolic disorders [14]. Similarly, the high prevalence of current or former smoking and regular alcohol use reflects global trends, where sustained exposure to these risk factors continues to drive disease burden [36]. Health behaviors in this age group are shaped by motivation, literacy, social support, and access to care [38,39].

Obesity, particularly central adiposity, remains a major concern due to its link with multiple chronic diseases [15]. The presence of extreme BMI and behavioral patterns in the sample underscores the diversity of risk among older adults, with some facing compounded threats from clustered behaviors. Finally, the overall positive self-rated health seen in the sample reflects a well-known trend: many older adults report high well-being despite chronic conditions [40]. While this optimism can support resilience and preventive engagement, it may also lead to the underestimation of health risks and lower motivation for change [13].

### 4.4. Positive Psychological Variables

Participants generally reported high levels of life satisfaction and a strong sense of meaning and purpose, alongside moderate optimism but lower self-esteem and self-efficacy. These patterns align with current research on psychological well-being in later life. Large-scale longitudinal studies highlight life satisfaction and optimism as key components of successful aging and overall quality of life [41,42]. For instance, Bhattacharyya et al. [41] found that life satisfaction not only predicts successful aging—measured through cognitive, physical, and social functioning—but that this relationship is reciprocal, with successful aging also reinforcing later-life satisfaction. Optimism was found to mediate this dynamic, helping individuals maintain well-being despite age-related challenges. Similarly, Cheng et al. [42] showed that optimism helps preserve life satisfaction in the face of increasing functional limitations. Even as older adults experience declines in daily functioning, those with a more optimistic outlook report significantly better well-being, reinforcing the role of optimism as a modifiable psychological asset.

In contrast, the lower levels of self-esteem and self-efficacy reported by participants reflect common declines associated with aging. Research by McAuley et al. [43] suggests that these traits are particularly vulnerable to reductions in physical activity and independence. Their study supports a hierarchical model in which diminished physical capability can erode self-perceptions of competence and worth. However, broader life satisfaction often remains resilient due to protective factors like optimism, a sense of meaning, and social connectedness, which help older adults adapt and maintain psychological well-being over time.

### 4.5. Negative Psychological Variables

The high prevalence of loneliness and social isolation in our sample reflects the emotional strain commonly experienced by individuals with chronic illnesses. For example, people living with hypertension often endure persistent stress related to fears of silent or undetected complications [16], while cancer survivors frequently report anxiety surrounding the possibility of recurrence [18]. Similarly, diabetes can contribute to social withdrawal due to the constant demands of self-management. However, this effect may be buffered in certain populations by protective factors such as strong family support or specific sample characteristics, including younger age cohorts [44]. Loneliness itself has physiological consequences—disrupting the hypothalamic–pituitary–adrenal (HPA) axis and increasing systemic inflammation—creating a biological link between social isolation and physical health risks, even in the absence of clinical depression [19].

The elevated levels of cynical hostility and vigilance observed in the sample echo psychological patterns like “diabetes distress,” in which individuals facing complex and demanding self-care routines become hyperaware of potential threats or failures [17]. While these heightened emotional states may serve as short-term coping strategies, they can eventually contribute to physiological dysregulation, such as poor glycemic control or cardiovascular strain [19]. Hostility and anger are often more prominent in hypertension, where the unpredictability of complications like stroke may amplify frustration and perceived helplessness [45].

Reports of perceived discrimination in the sample align with minority stress theory, which posits that chronic exposure to bias and social stigma induces persistent psychological arousal and stress responses. This is comparable to the distress experienced by cancer survivors coping with body image concerns or social stigma related to their diagnosis [18]. Over time, such stress can impair cognitive processes like attention and memory, creating a self-reinforcing cycle that exacerbates psychological strain.

Interestingly, while depressive symptoms were relatively low overall, a notable number of participants reported experiencing hopelessness—a core component of depression. This may reflect a perceived loss of control, particularly among those managing chronic conditions like diabetes, where frustration over disease progression is common [17]. Even subclinical symptoms like hopelessness deserve attention, as they can reduce treatment adherence and negatively impact long-term health outcomes [35].

The link between diabetes and depressive symptoms is particularly well-established. Meta-analyses indicate that individuals with diabetes are two to three times more likely to suffer from depression than those without the condition, largely due to the burdens of ongoing self-management, neuroendocrine disruption, and diabetes-specific emotional distress [17,46]. This relationship is bidirectional: depression can hinder effective glycemic control, while chronic hyperglycemia and insulin resistance promote neuroinflammation and neurotransmitter imbalances, thereby deepening depressive symptoms [46]. Feelings of hopelessness often stem from fears about complications such as neuropathy or retinopathy, which are prevalent among individuals with diabetes [47].

The significant association between diabetes and financial stress further underscores the multidimensional burden of the disease. Nearly 40% of nonelderly U.S. adults with diabetes report financial hardship due to medical costs, which often leads to nonadherence to treatment, food insecurity, and delayed care [48]. High costs related to insulin, glucose monitoring, and frequent medical appointments disproportionately impact low-income individuals, creating a cycle in which financial strain compromises self-care behaviors and worsens health outcomes [49].

In contrast, the psychological effects of a cancer diagnosis in our sample were less pronounced, with the only significant association emerging around chronic parental stress. Other psychological measures-including depression, hopelessness, and financial stress-did not reach statistical significance, despite some moderate to strong effect sizes. This suggests that, for most individuals in this sample, a cancer diagnosis may not significantly impair overall psychological well-being. However, chronic parental stress appears to be a distinct concern.

This aligns with the recent literature emphasizing the unique psychosocial toll that cancer can place on family dynamics, especially for parents. Research has shown that parents diagnosed with cancer often experience increased emotional distress and anxiety about their children’s well-being and adjustment [50,51]. Mothers with cancer, for example, report higher levels of stress and negative affect, which can impact their capacity to engage meaningfully with their children and maintain emotional availability [51]. Children, in turn, may struggle with worry, confusion, and a sense of emotional disconnection, further compounding family-wide stress [50]. This bidirectional strain likely accounts for the heightened levels of chronic parental stress observed among cancer patients in our sample.

Meanwhile, the significantly higher rate of cognitive impairment among individuals with hypertension is strongly supported by both longitudinal and cross-sectional studies. Hypertension disrupts cerebral blood flow, damages small vessels, and compromises the blood–brain barrier, leading to structural changes such as white matter hyperintensities, lacunar infarcts, and impaired neurovascular coupling, all hallmarks of vascular cognitive impairment [52,53,54]. These types of vascular injury are more directly linked to hypertension than to diabetes or cancer, which likely explains the absence of significant cognitive differences between those other groups in our sample.

Prospective studies have consistently shown that mid-life hypertension—defined as systolic blood pressure over 130 mmHg—is a key predictor of cognitive decline and dementia in later life [52,55]. Notably, hypertension is most strongly associated with impairments in attention, executive functioning, and language—cognitive domains governed by frontal and subcortical brain regions that are particularly vulnerable to vascular injury [55,56]. These patterns were reflected in the Cognitive Impairment Index, which likely captured deficits in these specific areas.

Crucially, the cognitive consequences of hypertension are distinct from those associated with diabetes or cancer. While diabetes does increase dementia risk, its cognitive effects are generally mediated through metabolic dysfunctions—such as insulin resistance and chronic hyperglycemia—rather than direct vascular injury [54,57]. In contrast, cancer-related cognitive decline is more often attributed to chemotherapy-induced neurotoxicity (“chemo brain”) and psychological stress rather than microvascular damage [18]. Hypertension, however, directly injures the brain’s small vessels, accelerates amyloid-β accumulation, and impairs glymphatic clearance, establishing a distinct and potent vascular pathway to cognitive decline [53,54].

Studies on the effects of anti-diabetic medication on cognitive function [6,11] offer valuable insight into possible mechanistic pathways linking metabolic disorders to neurocognitive health. In fact, GLP-1 agonists exert pleiotropic effects that link metabolic improvement to neurocognitive health by acting on anti-inflammatory, antioxidant, and insulin signaling pathways [6,11]. These mechanisms support their potential as disease-modifying therapies for neurodegenerative conditions, although further studies in humans are needed to confirm direct cognitive benefits [6,11].

### 4.6. Lifestyle and Hypertension, Diabetes, and Cancer

The data indicates that individuals with hypertension report a range of less healthy behaviors and outcomes compared to those without the condition. Specifically, they rate their health more poorly, have higher body weight and BMI, and engage in less physical activity. These patterns are consistent with existing research, which identifies excess weight and physical inactivity as major risk factors for both the development and progression of hypertension, as well as contributors to its associated comorbidities and complications [54]. Ungvari and colleagues [54] emphasize that obesity and sedentary behavior are particularly prevalent among people with hypertension, exacerbating vascular dysfunction and increasing the risk of cognitive decline.

Sleep disturbances also appear to be more common in individuals with hypertension. Although total sleep duration was similar across groups, those with hypertension reported more frequent difficulties falling and staying asleep. This aligns with research showing that poor sleep quality-including insomnia-is common in hypertensive populations and can disrupt blood pressure regulation, promote neuroinflammation, and elevate the risk of cognitive impairment [54].

The data also revealed a nuanced pattern of alcohol use. Participants with hypertension reported drinking more heavily per occasion and consuming more drinks per day, yet they drank on fewer days per month. This suggests a tendency toward binge or heavy episodic drinking, a pattern that is particularly harmful to vascular health. Ungvari et al. [54] note that even when overall alcohol frequency is not elevated, heavy drinking can exacerbate vascular injury and worsen hypertension-related outcomes.

Interestingly, no significant differences emerged between hypertensive and non-hypertensive participants in terms of fruit and vegetable consumption or smoking behavior. This suggests that these specific lifestyle factors may not distinguish between groups in this sample. These findings are consistent with broader research showing that, despite public health campaigns, adherence to healthy dietary patterns and smoking cessation remains suboptimal across the general population-including among individuals with hypertension [54].

Similarly, participants with diabetes reported a cluster of less healthy behaviors and outcomes relative to non-diabetic individuals: they rated their health more poorly, had higher body weight and BMI, and were less physically active. These findings are well-supported by the literature, which consistently identifies elevated BMI and low physical activity as both risk factors for the onset of type 2 diabetes and as common issues among those already diagnosed [58,59,60]. Obesity combined with physical inactivity not only increases the risk of developing diabetes but also contributes to poorer disease management and long-term health outcomes [58,59].

The lower self-rated health among individuals with diabetes is similarly well-documented. Poor or fair self-assessed health is not only predictive of diabetes onset but also correlates with lower medication adherence, greater difficulty in managing the condition, and a higher risk of complications [60].

Sleep quality, though not duration, also differed by diabetes status. Participants with diabetes reported more frequent difficulties falling asleep, a pattern that reflects existing findings showing that poor sleep quality is linked to worse glycemic control and increased likelihood of diabetes complications—even when total sleep time is similar [58]. A similar pattern emerged in terms of alcohol use: while individuals with diabetes reported drinking on fewer days per month, they consumed more alcohol on each occasion. This suggests a pattern of heavier, less frequent drinking, which has been linked to poor glycemic control and elevated risk for diabetes-related complications [60].

As with the hypertension group, no significant differences were observed between diabetic and non-diabetic participants in terms of fruit and vegetable intake or smoking behavior. This again highlights the widespread challenge of adopting and maintaining recommended dietary and lifestyle habits, regardless of chronic disease status [58,60].

In contrast, the data suggest that a cancer diagnosis had a minimal impact on most of the health and lifestyle variables examined. Participants with and without cancer reported similar levels of fruit and vegetable consumption, sleep duration, weight, smoking, and alcohol use. The only significant difference was in self-rated health, with cancer survivors perceiving their health as slightly worse. However, this interpretation should be made cautiously due to the smaller sample size of participants with cancer, which may reduce statistical power.

These findings are consistent with studies showing that many cancer survivors maintain health behaviors similar to those of individuals without a cancer diagnosis. For example, large-scale cohort studies have found that a cancer diagnosis rarely prompts substantial changes in fruit and vegetable intake, except in specific subgroups such as patients with advanced-stage breast cancer [61]. Likewise, meta-analyses have found no consistent relationship between sleep duration and cancer incidence or survival, with no clear dose–response effect across most cancer types [62].

While some cancers and their treatments can cause significant weight loss due to inflammation, metabolic disruption, or side effects, this is not a universal finding—particularly in community-based samples [63]. In terms of smoking and alcohol use, research suggests that, although some survivors reduce tobacco use post-diagnosis, many continue to smoke, and their overall alcohol consumption often remains similar to that of the general population [64,65].

The observed decline in self-rated health among cancer survivors is well-supported by the literature. This subjective measure is influenced by a range of factors, including comorbid conditions, functional limitations, and psychological well-being-domains frequently affected by cancer and its treatment [66]. Even when objective health indicators are similar, cancer survivors are more likely to rate their health as fair or poor, possibly due to lingering symptoms, a fear of recurrence, or the broader psychological aftermath of facing a life-threatening illness.

### 4.7. Sociodemographic, Clinical, Behavioral, and Psychological Predictors of Hypertension, Diabetes, and Cancer

The logistic regression findings offer valuable insights into the sociodemographic predictors of hypertension, diabetes, and cancer, aligning with and extending the existing literature. For hypertension, age emerged as a consistent predictor, with each additional year associated with increased odds. This is in line with global evidence showing that blood pressure tends to rise with age due to vascular stiffening and accumulated cardiovascular risk factors [10]. The elevated odds among widowed individuals may reflect the well-documented link between social isolation, grief-related stress, and cardiovascular health deterioration. Literature has shown that widowhood can increase psychological stress and reduce social support, both of which are associated with poorer blood pressure regulation [16]. Similarly, the high odds observed among retired individuals are consistent with findings that retirement can coincide with reduced physical activity and increased sedentary behavior, both contributing to a higher hypertension risk [22]. Although female sex showed a slight increase in odds, it was not statistically significant, consistent with the mixed findings in the literature, where gender effects often interact with age and hormonal factors [67].

Regarding diabetes, the finding that females had significantly lower odds than males supports previous studies indicating sex-based differences in diabetes risk, possibly due to hormonal factors and fat distribution patterns [7]. The elevated risk among separated individuals is noteworthy and aligns with studies suggesting that marital disruption can lead to psychological distress and unhealthy behaviors, both of which are linked to impaired glucose regulation [17]. Although the association of widowed status approached significance, it did not reach conventional thresholds, suggesting a potential trend worth further investigation. The model’s modest explanatory power is typical in social and health research, where many unmeasured lifestyle and genetic factors also contribute to diabetes risk [68].

For cancer, the finding that age was positively associated with cancer diagnosis is in line with epidemiological trends showing increased cancer incidence with age [8]. The significantly elevated odds among those “keeping house” suggests that domestic inactivity or related socioeconomic factors may influence cancer risk, potentially through reduced screening or access to healthcare [69].

Overall, while the models demonstrate adequate fit, the relatively low Nagelkerke R² values across all three outcomes (6–11%) highlight that sociodemographic factors alone explain only a small portion of the variance in chronic illness. This reinforces the importance of including behavioral, environmental, and biological variables in future models. These findings support the literature’s emphasis on tailored, multifactorial approaches to chronic disease prevention and management [70].

Over half of the population has hypertension, linked to poor health, sleep issues, higher BMI, and alcohol use. Interventions should focus on education about diet, medication adherence, blood pressure monitoring, low-impact exercise for older adults, sleep hygiene counseling, and support for reducing alcohol intake [71]. About 28% are obese, with some extreme cases, needing personalized nutritional counseling, combined weight management programs, and the monitoring of outliers for clinical follow-up. Smoking affects 16.6% of the study sample, including many daily smokers with a low use of cessation aids. Programs should increase access to cessation aids, offer counseling for relapse prevention, and integrate with chronic disease care [72].

Mental health issues include low self-esteem, loneliness, hostility, and moderate service use. The recommended actions include cognitive behavioral therapy, social group programs, self-efficacy building, stress management, and regular depression screening, especially for diabetics [73]. Chronic illness support should address depression and financial stress in diabetics with integrated care, family counseling for cancer survivors, and cognitive training plus caregiver support for cognitive impairment, especially in hypertensive patients [74]. Physical activity is below average in about a third of people; tailored exercise programs for older adults, including strength and balance training, plus community or home-based options are needed [75]. Finally, social and environmental challenges like discrimination require culturally sensitive care, stigma reduction through community education, and stronger social support networks [76].

The logistic regression results across multiple models offer valuable insights into how healthcare utilization, health behaviors, and psychological factors relate to the likelihood of chronic disease diagnoses, including hypertension, diabetes, and cancer. Starting with healthcare utilization and hypertension, the model significantly outperformed the null but explained only a modest proportion of the variance. Hospital admissions emerged as a weak yet significant positive predictor, consistent with prior research suggesting that comorbid conditions often co-occur with increased health service use [77]. Conversely, emergency room and primary care visits were negatively associated with hypertension diagnosis, a somewhat counterintuitive finding that may reflect underdiagnosis in more chaotic or episodic care environments [78] or potentially delayed diagnoses among individuals who engage with the healthcare system more frequently for other issues.

The diabetes model failed to achieve statistical significance overall, and none of the individual predictors—including hospital admissions or outpatient visits—reached significance. While the model fit was acceptable, its predictive value was weak, aligning with literature that points to the complex, multifactorial causes of diabetes, many of which lie outside of typical healthcare utilization patterns [79]. Similarly, the cancer model offered minimal explanatory power, and none of the healthcare utilization variables were significantly associated with diagnosis. This aligns with findings that many cancers remain asymptomatic and undetected until routine screenings or late-stage symptom presentation [80], limiting the utility of health service frequency as a predictive tool.

In contrast, health behavior models showed stronger associations. For hypertension, self-rated health, BMI, and physical activity were all significant predictors, which is consistent with the substantial body of literature linking poor perceived health and obesity to higher hypertension risk [81]. Physical activity slightly lowered the odds of diagnosis, echoing findings from the National Health Interview Survey and other epidemiological data [82]. For diabetes, the most powerful predictors were again BMI and self-rated health, with the model explaining up to 21.7% of the variance, substantially more than the healthcare utilization models. This is in line with the established risk factors for type 2 diabetes, where BMI is one of the most robust predictors [83].

The cancer behavior model showed that worse self-rated health was associated with higher odds of diagnosis (OR = 4.576), a finding that aligns with the literature [66]. Additionally, higher fruit and vegetable consumption was linked to reduced odds of diagnosis, supporting dietary recommendations in cancer prevention guidelines [84].

Turning to psychological factors, the models revealed complex and sometimes contradictory relationships. For hypertension, higher life satisfaction was associated with lower risk, aligning with the literature linking positive affect to reduced cardiovascular risk [85]. Also, the finding that depressive symptoms were also negatively associated with hypertension contradicts many studies reporting a positive association [86]. This discrepancy could stem from sample-specific differences or potential confounding effects. For diabetes and cancer, lower life satisfaction and optimism were associated with increased risk; also, depressive symptoms were linked to lower risk. These patterns are in line with conventional assumptions [87].

Taken together, the findings suggest that health behaviors are more reliable predictors of chronic illness than healthcare utilization patterns or psychological factors, at least in terms of statistical significance and variance explained. These results reinforce the importance of lifestyle interventions and behavior-based risk assessments in chronic disease prevention.

### 4.8. Practical Implications for Clinical and Community-Based Interventions

The findings of this study have several practical implications for clinical and community-based interventions targeting older adults with chronic conditions such as hypertension, diabetes, and cancer. First, the strong relationship between chronic illness and modifiable health behaviors highlights the need for tailored lifestyle interventions within primary care and community health settings. Programs focusing on physical activity promotion, dietary improvements, sleep hygiene, and alcohol moderation should be prioritized, particularly for individuals with hypertension and diabetes, who reported higher BMI, lower physical activity, and more sleep disturbances. These interventions should also account for the psychosocial barriers-like low self-efficacy and perceived hopelessness-that hinder behavior change. Integrating behavioral counseling and motivational interviewing into routine chronic disease management could help bridge the gap between health knowledge and consistent action, especially in populations with lower psychological resources.

Second, the psychological findings underscore the importance of incorporating mental health support into chronic illness care models. While older adults reported generally high life satisfaction and purpose, lower self-esteem, prevalent loneliness, and disease-specific stress (such as parental stress among cancer survivors or hostility in hypertension) indicate that emotional and cognitive burdens remain under-addressed. Interventions should target not only clinical depression but also subclinical distress like hopelessness, social isolation, and perceived discrimination. Expanding access to geriatric mental health services—through telehealth, peer-support networks, and integrated care teams—could mitigate the emotional toll of chronic illness. Furthermore, screening for cognitive impairment among hypertensive patients should be routine, given its strong link to vascular damage and long-term functional decline. By coordinating physical, cognitive, and emotional health strategies, clinicians and community providers can more effectively support aging adults in managing chronic diseases and maintaining quality of life.

### 4.9. Limitations

This study is subject to several methodological limitations that should be considered when interpreting its findings. Firstly, its cross-sectional design precludes any conclusions about causality, as it captures data at a single point in time rather than tracking changes or developments over time [88,89]. Secondly, there is a potential for self-selection bias, as the individuals who chose to participate may differ systematically from those who did not, possibly skewing the results. Furthermore, the sample includes individuals with only one chronic disease, which differs slightly from real-world patterns, where patients often experience two or more chronic conditions simultaneously. Also, the study lacks disease-specific measures, which limits the ability to draw precise conclusions about the unique impacts or needs associated with particular health conditions, thereby reducing the specificity and applicability of the findings to distinct patient populations. Furthermore, the reliance on self-reported data for both health behaviors and psychological indices introduces the potential for recall bias and social desirability bias, which may affect the accuracy of responses [88]. Third, while the sample is representative of older adults, it may not fully capture the experiences of the very elderly or those from more diverse cultural or socioeconomic backgrounds, limiting the generalizability of the results [90]. Fourth, this study utilizes secondary data exclusively from the United States. As a result, the findings may not be directly generalized to other countries or regions with different social, economic, or cultural contexts. However, it is important to note that using geographically specific data is a common practice in many studies, and such data still provide valuable insights that can inform future research and policy both within and beyond the context studied. Moreover, another limitation concerns the fact that the Wave 6 [29] sample had already responded to previous versions (prior waves). In fact, whether the participants reported the same data multiple times may have introduced some bias. Additionally, the study did not explore the nuanced impacts of multimorbidity or the specific effects of combinations of chronic conditions, which are common in aging populations and may influence both physical and psychological health in complex ways [68,91]. Finally, although the study included a range of health and psychological variables, it did not assess social well-being or the indirect economic burden of chronic disease, both of which are increasingly recognized as important determinants of quality of life in older adults [68].

### 4.10. Future Research

Future research should employ longitudinal designs to clarify causal relationships between chronic illness, health behaviors, and psychological outcomes in older adults, as cross-sectional studies cannot determine directionality or long-term effects [89]. Expanding the focus to include social well-being, social support, and the impact of multimorbidity will provide a more comprehensive understanding of how these factors interact to influence health and quality of life [92,93]. Additionally, studies should include more diverse populations to improve generalizability and address health disparities. Finally, evaluating the effectiveness of integrated care models and targeted interventions—such as those promoting vascular health and cognitive resilience in hypertension—will help to inform strategies for healthier aging [54].

## 5. Conclusions

This study provides novel insights by systematically addressing gaps in chronic illness research, particularly the tendency to homogenize hypertension, diabetes, and cancer despite their distinct etiologies and psychosocial impacts. By analyzing these conditions side-by-side, the findings reveal different behavioral and psychological profiles that underscore the need for condition-specific interventions. For instance, hypertension’s strong association with cognitive impairment and specific drinking patterns (e.g., heavier episodic use) highlights its vascular pathophysiology, while diabetes’s ties to depressive symptoms and financial stress emphasize its metabolic and socioeconomic dimensions. Cancer’s link to parental stress—a previously underexplored outcome—exposes the familial burden often overlooked in cancer care. The observed association between cancer and heightened parental stress highlights the broader psychosocial impact of the disease beyond the individual patient. This finding suggests that cancer care should increasingly adopt a family-centered approach, recognizing parents not only as caregivers but also as individuals whose own well-being may be compromised. Integrating psychosocial assessments into oncology settings to screen for parental stress, particularly among those with dependent children, could help identify at-risk families early. Tailored interventions—such as family counseling, parenting support groups, or childcare resources—could then be offered to alleviate stress and enhance family resilience. Such support may improve both caregiver functioning and patient outcomes, as reduced parental stress can foster a more stable and supportive home environment during treatment and recovery.

This study advances the field by integrating both positive (e.g., life satisfaction, meaning) and negative (e.g., hostility, vigilance) psychological constructs, moving beyond the traditional focus on depression alone. The identification of both positive and negative psychological factors has important clinical implications for chronic disease management. Negative aspects such as distress, loneliness, and perceived burden were associated with poorer self-rated health, underscoring the need for psychological screening as part of routine chronic disease care. Addressing these through interventions like mental health referrals, social support programs, or stress-reduction strategies could mitigate their impact on disease outcomes. Conversely, positive psychological constructs—such as a sense of purpose, gratitude, or optimism—were linked to better health perceptions and may act as protective factors. Clinicians could leverage these strengths by incorporating positive psychology techniques into care plans, such as goal-setting based on personal values or gratitude journaling, which may enhance engagement and adherence. Recognizing and addressing this dual psychological profile allows for a more holistic, patient-centered approach that can improve both mental well-being and physical health outcomes in chronic disease populations.

This study also addresses gaps in health behavior research by examining nuanced patterns, such as how alcohol use diverges between hypertensive and diabetic individuals despite similar overall consumption levels. By leveraging a nationally representative sample of older adults—a group disproportionately affected by chronic disease—the findings challenge assumptions about uniform health behaviors across conditions, revealing that fruit/vegetable intake and smoking habits remain consistent regardless of diagnosis.

Individuals with hypertension, diabetes, or cancer tend to display distinct but occasionally overlapping demographic, behavioral, psychological, and healthcare-related characteristics. For hypertension, the likelihood increases with age, with odds rising by 3.7% for each additional year. Widowed individuals are significantly more likely to have hypertension compared to their married counterparts, with 2.4 times higher odds. Similarly, being retired is strongly associated with the condition, with retired individuals showing 7.6 times greater odds of having hypertension than those currently working. While females had slightly higher odds of hypertension compared to males, this difference was not statistically significant. Health service usage patterns revealed that individuals with more hospital admissions in the past year had higher odds of hypertension, while those who made more frequent visits to emergency rooms and healthcare professionals such as doctors or nurse practitioners had lower odds, potentially indicating underdiagnosis or less engagement among certain groups. Regarding health behaviors, higher BMI and poorer self-rated health were both associated with increased odds of hypertension, while more frequent physical activity was modestly protective. Psychologically, individuals reporting lower life satisfaction, greater depressive symptoms, and higher optimism paradoxically showed higher odds of hypertension, suggesting complex interactions between mental well-being and health outcomes. In the case of diabetes, sex and marital status played notable roles. Females had significantly lower odds of diabetes compared to males, with a 48% reduction in likelihood. Separated individuals were at substantially higher risk, showing over seven times the odds of diabetes compared to married people. Widowed status approached significance as well. Unlike with hypertension, age and employment status were not significant predictors. Healthcare utilization did not show meaningful associations, indicating that diabetes diagnosis might not be strongly linked to recent health service use. Similarly to hypertension, higher BMI and poorer self-rated health were significant contributors to increased diabetes risk, while physical activity was not a significant factor. From a psychological standpoint, those with lower life satisfaction, lower optimism, and greater depressive symptoms were more likely to have diabetes, reinforcing the connection between mental health and chronic disease.

By prioritizing condition-specific complexities and modifiable risk factors, this study lays the groundwork for tailored interventions, such as integrating routine cognitive screening into hypertension care using brief, validated tools like the Mini-Cog during annual check-ups or embedding financial counseling services within diabetes management programs to assist patients in navigating medication costs, dietary expenses, and insurance coverage. These targeted approaches could be operationalized through multidisciplinary care teams, electronic health record prompts, and community-based partnerships, ensuring that interventions are both scalable and responsive to the real-world challenges faced by patients with chronic conditions.

These findings not only fill gaps identified in the literature but also redefine how chronic illnesses are studied, advocating for precision public health strategies that account for the interplay of biology, behavior, and psychosocial context.

## Figures and Tables

**Table 1:** Clinical variable frequencies.

Variable	Yes (%)	No (%)
Hypertension	56.1%	43.9%
Diabetes	20.8%	79.2%
Cancer	19.9%	80.1%

**Table 2:** Clinical service use frequencies.

Variable	Mean	Median	Standard Deviation
Hospital Admissions	0.63	0.00	3.38
Emergence Room Visits	0.61	0.00	1.13
Health Professional Visits	6.00	4.00	9.41
Mental Health Visits	4.59	5.00	1.22

**Table 3:** Health behavior variables.

Variable	Key Statistics	Notes
1. Self-rated health	40% “Very good,” 29.3% “Good,” 4% “Poor”	Most respondents rate health positively
2. Fruit/vegetable servings per day	44% eat 1–2 servings, 39.7% eat 3–4 servings, 1.9% eat none	Low adherence to recommended intake (5+ servings: 14.5%)
3. Sleep duration (hours)	Mean: 7.29 h; 31.8% sleep 8 h, 26.7% sleep 7 h	Moderate variability (SD: 1.545), extreme values (2–19 h)
4. Sleep duration (minutes)	95.7% report 0 min (likely indicating rounding to whole h)	Only 4.2% added minutes (e.g., 30 min)
5. Frequency of trouble falling asleep	53.2% “Rarely/never,” 31% “Sometimes,” 6.1% “Almost daily”	15% experience frequent issues
6. Frequency of waking up and struggling to sleep	36.2% “Rarely/never,” 42.9% “Sometimes,” 7.2% “Almost daily”	20.8% report frequent disruptions
7. Weight (kg)	Mean: 181.15 kg (SD: 44.7); range: 77–350 kg	Extreme values suggest data entry errors
8. Ever smoked	55.4% “Yes”	Over half are former smokers
9. Smoked > 100 cigarettes in lifetime	99.4% “No”	Limited to small subgroup (likely current/former smokers)
10. Current smoker	16.6% “Yes”	Majority (83.4%) do not smoke
11. Smoking days per week	Mean: 6.34 days (SD: 1.49); 81.7% smoke daily	Most smokers smoke every day
12. Cigarettes/packs per day	Mean: 5.64 (SD: 5.76); 14.3% smoke 10+ cigarettes	Heavy smoking is rare
13. Unit of cigarettes per day	68.6% report “cigarettes,” 31.4% “packs”	Reflects measurement preferences
14. Ever tried to quit smoking	77.6% “Yes”	Majority of smokers attempted to quit
15. Used products/services to quit smoking	21% “Yes”	Low utilization of cessation aids
16. Drinks alcohol	61.6% “Yes”	Majority consume alcohol
17. Drinking days per month	Mean: 7.89 days (SD: 9.6); 19.5% abstained last month	Wide variability (0–31 days)
18. Drinks per occasion	Mean: 1.84 drinks (SD: 1.64); 50.1% have 1 drink, 30.5% have 2 drinks	Moderate alcohol intake per session
19. Body Mass Index (BMI)	Mean: 28.54 (SD: 6.28); 28.2% obese (BMI ≥ 30), 4.2% underweight (BMI < 18.5)	High obesity rates; extreme values (BMI 13–57) require scrutiny
20. Physical Activity Index, standardized (3 items)	Mean: −0.12 (SD: 1.033); median: −0.10; range: −2 to +1; frequency: −2: 7.2%; −1: 25.5%; 0: 25.6%; +1: 13.5%	Scores standardized (likely z-scores) with negative values indicating below-average activity Majority cluster near mean (−0.12) but 13.5% report higher activity (+1)

**Table 4:** Positive psychological variables.

Variable	Mean	Median	Standard Deviation
Life Satisfaction	2.20	2.00	0.81
Optimism Index	3.10	3.00	0.64
Self-Esteem Index	−1.08	−1.17	0.57
Meaning, Potential, and Generativity Index	1.51	1.40	0.54
Self-Efficacy Index	−1.39	−1.24	0.64

**Table 5:** Negative psychological indicators.

Variable	Mean	Median	Standard Deviation
Loneliness Index	3.10	3.00	1.12
Chronic Parental Stress Index	−0.19	−0.22	1.13
Cognitive Impairment Index	0.55	0.00	1.36
Depressive Symptoms (CESD-11)	−0.21	−0.18	1.17
Anger-Out Index	1.37	1.33	0.72
Cynical Hostility Index	2.45	2.33	0.95
Hopelessness Index	1.79	2.00	1.01
Everyday Discrimination Index	1.66	1.60	1.00
Vigilance Index	2.60	2.60	0.82
Chronic Financial Stress Index	−0.40	−0.46	1.20

**Table 6:** Associations between hypertension diagnosis and health behavioral variables.

Health Behavior	Hypertension (Yes) Mean (SD)	Hypertension (No) Mean (SD)	*t*	*p*-Value	Cohens’ *d*
Self-Rated Health	2.77 (0.987)	2.28 (0.966)	6.726	<0.001	0.506
Problems Falling Asleep	1.78 (0.929)	1.59 (0.794)	2.921	0.004	0.222
Problems Waking Up and Getting Back to Sleep	1.97 (0.911)	1.82 (0.811)	2.338	0.020	0.178
Weight	187.17 (48.468)	175.91 (38.192)	3.367	<0.001	0.254
Alcohol Consumption	2.67 (1.974)	2.36 (1.897)	2.102	0.036	0.158
Days Drinking Alcohol Per Month	6.79 (8.897)	8.94 (10.145)	−2.352	0.019	−0.225
Number of Drinks Per Day	2.00 (2.167)	1.63 (0.921)	2.092	0.037	0.223
Body Mass Index (BMI)	29.49 (6.717)	27.65 (5.524)	3.932	<0.001	0.296
Physical Activity Index	−0.28 (1.045)	0.09 (0.968)	−4.859	<0.001	−0.370

**Table 7:** Associations between diabetes diagnosis and health behavioral variables.

Variable	Diabetes Status	N	Mean	Standard Deviation	*p*-Value
Self-rated health	Yes	142	3.10	0.96	<0.001
	No	542	2.41	0.98	
Problems falling asleep, last 4 weeks	Yes	139	1.83	0.91	0.029
	No	530	1.65	0.86	
Weight	Yes	142	190.35	43.79	0.012
	No	536	179.86	44.44	
Drinking alcoholic beverages	Yes	142	3.00	2.01	<0.001
	No	541	2.38	1.90	
Days drinking beer, wine, or liquor	Yes	70	4.89	7.20	0.005
	No	351	8.36	9.81	
Body Mass Index (BMI)	Yes	142	29.56	6.13	0.049 *
	No	539	28.42	6.25	
Physical Activity Index	Yes	139	−0.43	1.04	<0.001
	No	530	−0.03	1.00	

* *p* = 0.051 for equal variances assumed, *p* = 0.049 for unequal variances assumed.

## Data Availability

The data used in this study are public after authorization is requested for research purposes.
